# Supplementary material for: Low-Dose SARS-CoV-2 S-Trimer with an Emulsion Adjuvant Induced Th1-Biased Protective Immunity
Source: Int J Mol Sci. 2022 Apr 28;23(9):4902. doi: 10.3390/ijms23094902 (PMC9101745; doi:10.3390/ijms23094902)
Supplement: Supplementary file 1 [file ijms-23-04902-s001.zip › ijms-1680084-supplementary.pdf]

International Journal of *Molecular Sciences*

MDPI

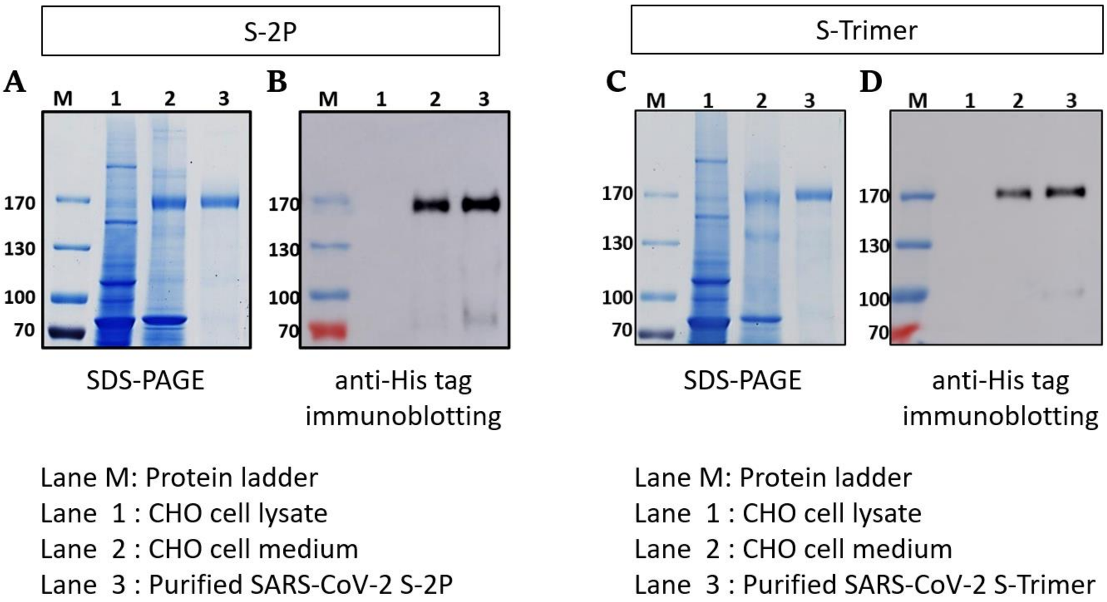

Figure S1. Expression and purification of modified SARS-CoV-2 spike proteins

Production of SARS-CoV-2 S-2P and S-trimer was monitored by (**A**,**C**) SDS–PAGE analysis and (**B**,**D**) immunoblotting with anti-His-tagged antibodies. Lane M: molecular weight ladder, with relevant bands labeled in kilodaltons; Lane 1: transient expressing CHO cells diluted in PBS; Lane 2: filtered medium supernatant from transfected CHO cells; Lane 3: elution of purified S protein from Ni-NAT affinity resin.

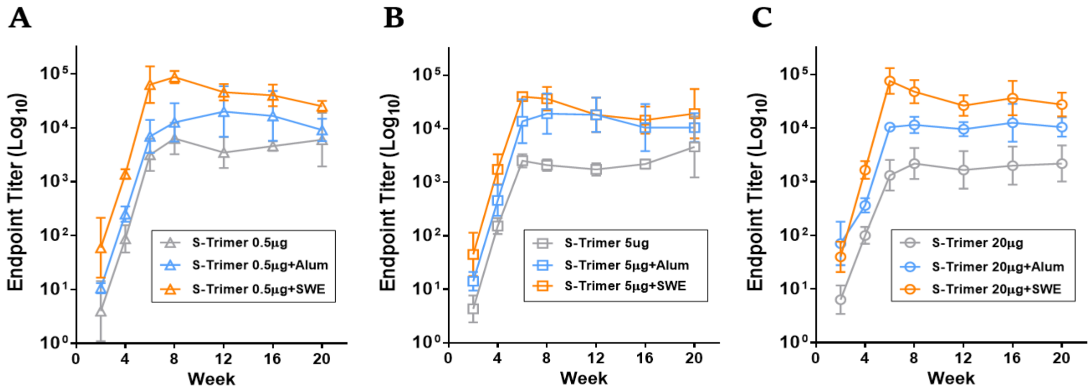

Figure S2. Antibody response induced by vaccination of S-trimer with human adjuvants

BALB/c mice (n = 5/ group) were intramuscularly immunized twice at a 4-week interval with (**A**) 0.5 µg or (**B**) 5 µg or (**C**) 20 µg of S-trimer protein with or without adjuvant (Alum or SWE). Serum samples were collected at the indicated time points after the first immunization. Endpoint titers (Log10) of the total IgG antibodies against the SARS-CoV-2 S-trimer protein was evaluated by ELISA.

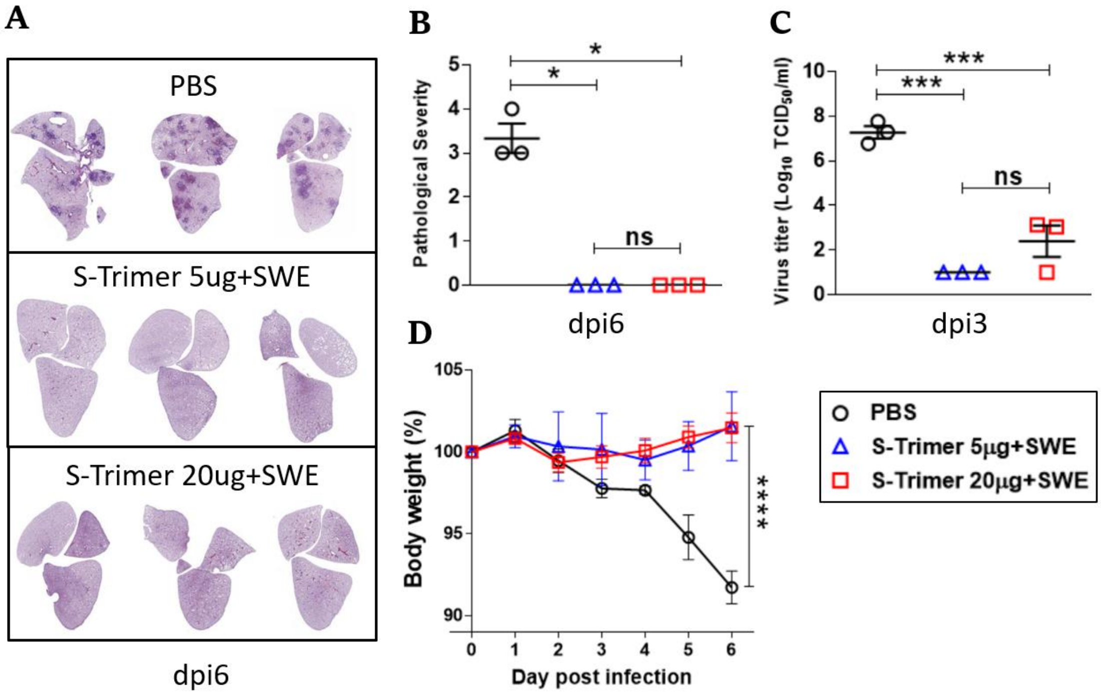

Figure S3. Immunization of S-trimer with SWE provides robust protection against SARS-CoV-2 infection in Syrian hamster

Syrian hamsters (n=6/group) were immunized with SWE-adjuvanted S-trimer at 5 μg or 20 μg twice via the intramuscular route on Days 0 and 28. The PBS-injected group was the blank control. Syrian hamsters were intranasally infected on Day 45 with 1×10^5 TCID50 live SARS-CoV-2 virus. (**A**) Bright field imager of H&E staining of lung sections from infected hamsters at dpi6, in which purple indicates areas of inflammation. (**B**) The pathological severity of lung lesions was evaluated by the percentage of inflammation area of each section. (**C**) Virus titers in the lungs of SARS-CoV-2-infected hamsters at dpi3 were determined by TCID50 assay. (**D**) Body weight change (%) of the hamsters was recorded every day after SARS-CoV-2 infection. Symbols represent individual animals; horizontal lines indicate the mean ± SD. Statistically significant differences were compared by a two-tailed Mann–Whitney test. The comparisons of body weight change (%) were made by two-way ANOVA with multiple comparisons tests. *P < 0.05, **P < 0.008, ***P < 0.0005, *ns*: not significant.
